# The Date Palm Tree Rhizosphere Is a Niche for Plant Growth Promoting Bacteria in the Oasis Ecosystem

**DOI:** 10.1155/2015/153851

**Published:** 2015-03-19

**Authors:** Raoudha Ferjani, Ramona Marasco, Eleonora Rolli, Hanene Cherif, Ameur Cherif, Maher Gtari, Abdellatif Boudabous, Daniele Daffonchio, Hadda-Imene Ouzari

**Affiliations:** ^1^LR03ES03 Laboratoire Microorganismes et Biomolécules Actives, Faculté des Sciences de Tunis, Université de Tunis El Manar, Campus Universitaire, 2092 Tunis, Tunisia; ^2^Biological and Environmental Sciences and Engineering Division, King Abdullah University of Science and Technology, Thuwal 23955-6900, Saudi Arabia; ^3^Department of Food, Environment, and Nutritional Sciences, University of Milan, Via Celoria 2, 20133 Milan, Italy; ^4^Université de La Manouba, Institut Supérieur de Biotechnologie de Sidi Thabet, LR11ES31 LR Biotechnologie & Valorisation des Bio-Géo Ressources, BiotechPole Sidi Thabet, 2020 Ariana, Tunisia

## Abstract

In arid ecosystems environmental factors such as geoclimatic conditions and agricultural practices are of major importance in shaping the diversity and functionality of plant-associated bacterial communities. Assessing the influence of such factors is a key to understand (i) the driving forces determining the shape of root-associated bacterial communities and (ii) the plant growth promoting (PGP) services they provide. Desert oasis environment was chosen as model ecosystem where agriculture is possible by the microclimate determined by the date palm cultivation. The bacterial communities in the soil fractions associated with the root system of date palms cultivated in seven oases in Tunisia were assessed by culture-independent and dependent approaches. According to 16S rRNA gene PCR-DGGE fingerprinting, the shapes of the date palm rhizosphere bacterial communities correlate with geoclimatic features along a north-south aridity transect. Despite the fact that the date palm root bacterial community structure was strongly influenced by macroecological factors, the potential rhizosphere services reflected in the PGP traits of isolates screened *in vitro* were conserved among the different oases. Such services were exerted by the 83% of the screened isolates. The comparable numbers and types of PGP traits indicate their importance in maintaining the plant functional homeostasis despite the different environmental selection pressures.

## 1. Introduction

The southern regions of Tunisia are very arid and the date palm (*Phoenix dactylifera* L.) is a key plant determining in the oasis agroecosystem a microclimate that favours agriculture [1]. The palms protection provides many ecosystem services, including ameliorating oasis temperature, changing floodwater dynamics and facilitating wildlife, and making agriculture possible under harsh environmental conditions [2]. In the world, oases cover about 800 000 ha and support the living of 10 million people. In Tunisia more than four millions of date palm trees are spread onto 32 000 ha of oasis in the southern part of the country [3, 4]. As a result of the oases overexploitation and strong anthropogenic pressures, these ecosystems are becoming increasingly fragile. Furthermore, despite the oasis potential to tolerate several abiotic stresses typical of arid environment, the ongoing climate change is enhancing the environmental pressure on the date palm affecting growth and development, especially in the Middle East [5].

Besides the well-known plant growth promoting properties typical of rhizospheres in temperate soils in nonarid ecosystems, rhizosphere bacteria in arid soils contribute in counteracting drought and salinity stresses, by providing services such as, among others, physical protection of the root from mechanical stress against the dry soil particles, induction of plant physiological responses against water losses [6], or productions of metabolites contributing to the maintenance of the plant hormone and nutrient homeostasis, [7]. In particular PGP (plant growth promoting) bacteria, naturally associated with plants, have been shown to be essential partners for improving plant tolerance to stressful conditions [8]. The exploration of plants naturally adapted to extreme condition may allow a reservoir of biodiversity exploitable to understand the ecological service enclosed in these ecosystems [8, 9]. In this context, ecological niche presented in the oasis ecosystem could provide a new model to study and dissect the key factors driving the stability of this ecosystem [10]. Little information is available about the microbiological functionality of both oasis and date palm. For instance the potential PGP services provided by the root-associated bacteria appear to be invariant with respect to geoclimatic factors despite provided by different bacterial communities, according to observations across a north to south aridity transect that included Tunisia [11].

Since plants contribute to shape soil microbial diversity [12, 13], the aim of this work was to assess bacterial communities associated with the date palm rhizosphere soil, the root surrounding soil and the bulk soil fractions in seven Tunisian oases, in order to evaluate if along a north-south transect (i) the assemblage of bacterial communities in the palm root soil fractions was driven by the geoclimatic factors and (ii) the ecological services were preserved in the soil fractions of the root system. The structure of the bacterial communities associated with the soil fractions of date palm in the seven oases was dissected by 16S rRNA gene-based PCR-DGGE (denaturing gradient gel electrophoresis) analysis. The results were analysed in function of geoclimatic factor and oasis origin, and compared with the diversity of the cultivable bacteria and their PGP potential.

## 2. Materials and Methods

### 2.1. Site Description and Sampling

**Figure 1:**
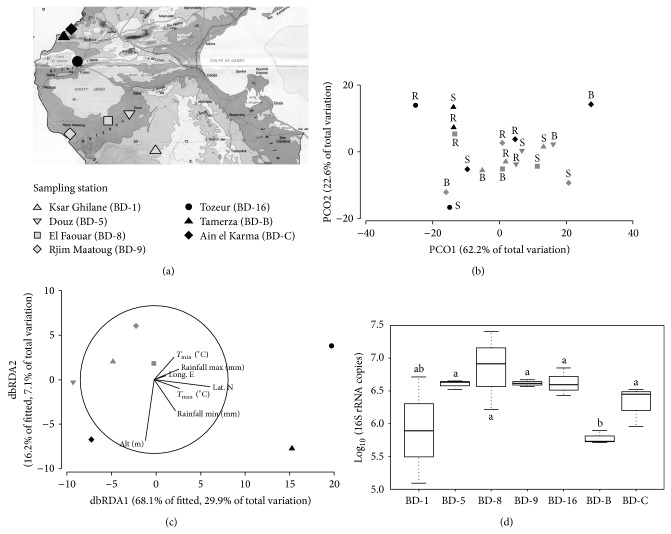
Station location and analysis of bacterial community structure associated with soil fraction of date root system. (a) The location of the studied oases is indicated on the map of Tunisia. (b) A principal coordinate analysis (PCO), deduced from the 16S rRNA gene-based PCR-DGGE profiles, resumes the diversity of the bacterial communities associated with the date palm root soil fractions. 84.8% of total variation is explained in the presented PCO. The soil fractions analysed are R: rhizosphere; S: root surrounding soil; and B: bulk soil. (c) Dist LM analysis to evaluate which are the main geoclimatic variables influencing the structure of the bacterial communities associated with date palm root soil fractions. Lat. N: latitude north; Long. E: longitude east; Alt.: altitude; *T*_min⁡_: minimum temperature; *T*_max⁡_: maximum temperature; Rainfall min: minimum rainfall; Rainfall max: maximum rainfall. (d) Box-plot graph represents the quantification of 16S rRNA gene by qPCR. The number of copies is expressed in Log_10_. Statistical analysis (pairwise test) of bacterial assemblage across locations was indicated by the letter.

The sampling was carried out from seven oases in different geographic locations in Tunisia, along a latitude/longitude gradient, respectively from 32° to 34°N and from 7° to 9°E (Figure 1(a) and Supplementary Table 1 in the Supplementary Material available online at http://dx.doi.org/10.1155/2015/153851). A traditional crop management was used in all the oases, including groundwater-based flooding irrigation and fertilization with organic fertilizers. In each oasis, the roots of three date palm trees of similar age, lying in the distance of less than 15 meters and growing in the same soil were separately collected at 20–30 cm depth in order to obtain the adhering rhizosphere soil (R) tightly attached to roots. After removing the roots, the root surrounding soil (S) was collected. Bulk soil samples (B) not influenced by date palm root system were also sampled as control. All soil samples were collected under sterile conditions using sterile tools. Recovered samples were stored at −20°C for molecular analysis or at 4°C for isolation.

### 2.2. Total DNA Extraction, PCR-DGGE, and Profile Analysis

Total DNA from soil samples was extracted by commercial kit FASTDNA SPIN KIT for soil (Qbiogene, Carlsbad, USA) according to the manufacturer's procedure. PCR amplification was performed in a final volume of 50 *μ*L using primers 907R and 357F, adding a GC-clamp to the forward primer [14, 15]. The reaction mixture was prepared with 1X PCR buffer, 2.5 mM MgCl_2_, 0.12 mM deoxynucleoside triphosphate, 0.3 mM of each primer and 1U Taq DNA polymerase and 10 ng of pooled DNA obtained from the three plant replicates were added as template. PCR products were resolved on 7% (w/v) polyacrylamide gel in 1X TAE pH 7.4 with a 40–60% denaturing gradient. Gels were run at 90 V for 17 h at 60°C in DCode apparatus (Bio-Rad, Italy). After electrophoresis, gels were stained with ethidium bromide solution for 30 min, washed with sterile distilled water, and photographed on a UV transillumination table. The DGGE band profiles were converted into numerical values using Image J (version 1.46) and XLSTAT software.

### 2.3. Real Time PCR

Quantitative real time PCR (q-PCR) was performed on a Chromo4 real time PCR machine (Bio-Rad) to measure the presence and concentration of bacterial 16S rRNA gene associated with the rhizosphere fractions. The reactions were performed with IQ SYBR Green Supermix (Bio-Rad), using primers targeting the 16S rRNA gene (Bac357-F and Bac907-R) [16]. PCR SYBR green reactions were prepared by using the “Brilliant SYBR Green QPCR Master Mix” kit (Stratagene) in 96-well plates. The reaction mix (25 mL) contained 1X Brilliant SYBR Green (2.5 mM MgCl_2_), 0.12 mM of each primers, and approximately 100 ng of extracted DNsA. The DNA obtained from the three plants sampled in the same station was pooled and used as template to carry out the real time assay in triplicate. At the end of each real time PCR, a melting curve analysis was performed for verifying the specificity of PCR products. To construct standard curves, the 16S rRNA gene of* Asaia* sp. was amplified by PCR and cloned using the pGEM T-easy Vector Cloning Kit (Promega). q-PCR data relative to the 16S rRNA gene concentration were log-transformed.

### 2.4. Isolation of Cultivable Bacteria

One gram of rhizosphere soil (R) from each sample was suspended in 9 mL of sterile physiological solution (9 g/L NaCl) and shaken for 15 min at 200 rpm at room temperature. Suspensions were diluted in tenfold series and plated in triplicate onto TSA (Tryptic Soy Agar), YEM (Yest Extract Mannitol), and KB (King'B agar) culture media. After three days at 30°C colonies were randomly selected and spread on the original medium for three times to avoid contamination risks. Pure strains were frozen in 25% glycerol at −80°C. A total of 440 isolates were collected. The isolates were named based on the station and the medium from which they were isolated.

### 2.5. DNA Extraction, Dereplication, and Identification of Isolates

Genomic DNA was recovered from the isolates using a boiling lysis. Bacterial cells were suspended in 50 *μ*L of sterile TE (10 mM Tris/HCl, pH 8, 1 mM EDTA) and incubated at 100°C for 8 min. After centrifugation (13000 g, 10 min), the supernatant containing the released DNA was stored at −20°C and used as template for PCR. Amplification of 16S–23S internal transcribed spacer region (ITS) was performed using the universal primers S-DBact-0008-a-S-20 (5′-CTA CGG CTA CCT TGT TAC GA-3′) and S-D-Bact-1495-a-S-20 (5′-AGA GTT TGA TCC TGG CTC AG-3′) according to the procedure described previously by Fhoula et al. [17]. Two *μ*L of the PCR products were checked by electrophoresis in 1.5% agarose gel and stained with ethidium bromide. Gel images were captured using Gel Doc 2000 system (Bio-Rad, Tunis, Tunisia), and bacteria redundancy was reduced by evaluating the different ITS profiles. One strain per each ITS haplotype was used in the phylogenetic analysis and for further experiments. A total of 98 strains were characterized by 16S rRNA gene sequencing using the primers S-D-Bact-1494-a-20 (5′-GTC GTA ACA AGG TAG CCG TA-3′) and L-D-Bact-0035-a-15 (5′-CAA GGC ATC CAC CGT-3′). PCR amplification was carried out as described by Fhoula et al. [17]. The 16S rRNA PCR amplicons were purified with Exonuclease-I and Shrimp Alkaline Phosphatase (Exo-Sap, Fermentas, Life Sciences) following the manufacturer's standard protocol. Sequencing of the purified amplicons was performed using a Big Dye Terminator cycle sequencing kit V3.1 (Applied Biosystems) and an Applied Biosystems 3130XL Capillary DNA Sequencer machine. The obtained sequences, with an average length of 750 bp, were compared with those available at the National Centre for Biotechnology Information (NCBI) database (http://www.ncbi.nlm.nih.gov) using the basic local alignment search tool (BLAST) algorithm [18]. The 16S rDNA sequences were submitted to the NCBI nucleotide database under the accession number KJ956590 to KJ956687.

Phylogenetic analysis of the 16S rRNA gene sequences was conducted with molecular evolutionary genetics analysis (MEGA) software, version 6 [19]. Trees were constructed by using neighbor-joining method [20].

### 2.6. Characterization of Plant Growth Promoting Activity and Abiotic Stress Resistance

The 98 bacterial strains identified were screened for production of indole acetic acid (IAA), siderophores and ammonia, mineral phosphate solubilization, protease and cellulose activity, and tolerance to several abiotic stresses. Quantitative production of IAA was determined as described by Ouzari et al. [21]. Briefly, after incubation in minimal medium supplemented with glucose (100 g/L) and L-tryptophane (10 *μ*g/mL), using Salkowski's reagent, the colour absorbance was read, after 20–25 min, at 535 nm. Concentration of IAA produced was measured by comparison with a standard graph of IAA. Ability of bacteria to solubilize inorganic phosphate was evaluated as described by Nautiyal [22], by the observation of clear halo around the bacterial colony grown in Pikovskaya medium. To demonstrate the production of siderophore, the tested strains were spotted on nutrient agar plates. After incubation for 48–72 h at 30°C, the grown strains were overlaid with CAS medium supplemented with agarose (0.9% w/v). Positive test was noted when colour modification around colonies from blue to orange was observed [23]. Ammonia production was assayed by inoculation of bacterial strains in 10 mL of peptone water and using Nessler's reagent (0.5 mL). Ammonia producing strains were identified when brown to yellow colour was developed [24]. Protease (casein degradation) and cellulase activities were determined by spot inoculation of the strains on Skimmed milk and CMC agar media, respectively. A clear halo around the colonies indicates the ability of the strains to produce the degrading enzymes [25].

Tolerance to osmotic stress was evaluated by adding to tryptic soy broth (TSB) medium 30% of polyethylene glycol (PEG 8000). Resistance to salt was assessed by adding 5, 10, 15, 20, 25, and 30% NaCl to the culture media and incubating the plates at 30°C for 5 days. The ability to growth at 45, 50, and 55°C was checked in TSA by incubation at the indicated temperatures for 5 days. Tolerance to acid (3 and 4) and basic (10 and 12) pH was assessed by adjusting the medium with concentrated HCl (12 N) and NaOH (3 M), respectively.

### 2.7. Statistical Analysis

Significant differences in soil bacterial community structure were investigated by permutational analysis of variance (PERMANOVA, [26]). Distance-based multivariate analysis for a linear model (DistLM, [27]) was used to determine which significant environmental variables explain the observed similarity among the samples. The Akaike information criterion (AIC) was used to select the predictor variables [28]. The contribution of each environmental variable was assessed: firstly the “marginal test” is used to assess the statistical significance and percentage contribution of each variable by itself and secondly the “sequential test” was employed to explain the biotic similarity considering all the variable contributions. All the statistical tests were performed by PRIMER v. 6.1 [29], PERMANOVA+for PRIMER routines [30].

## 3. Results and Discussion

### 3.1. Environment Parameters Directly Influence Bacterial Communities Associated with Palm Rhizosphere

The diversity of bacterial communities associated with the date palm root system from each of the seven studied oasis was investigated through the analysis of the diversity of the 16S rRNA gene in the rhizosphere (R) and root surrounding soil (S) fractions. Bulk soil (B) was also included as a comparative fraction not directly influenced by the plant root. Separation among palm bacterial communities located in north (BD-16, BD-B, and BD-C) and south (BD-1, BD-5, BD-8, and BD-9) oases was supported by a principal coordinate analysis (PCO) (Figure 1(b)), suggesting that geoclimatic conditions influence the bacterial community structure. Statistical analysis confirmed the grouping observed in the PCO analysis with a significant difference between north and south oases (PERMANOVA, df = 1.55; *F* = 8.06; *P* = 0.0017) but not a significant separation mediated by the aquifer used to irrigate the oases (PERMANOVA, df = 1.55; *F* = 1.45; *P* = 0.21).

Within the two macroregions, the north and south groups of oases, we observed a significant difference among oases (Supplementary Table 2) indicating the presence of oasis-specific bacterial community supporting a concept of “ecological island.” Pairwise analysis showed that such differences observed among the oases predominantly occurred in the north regions (Supplementary Table 3), possibly because the south region (closest to desert) presents harsher conditions that select a more restricted type of bacteria. These ecological islands represent specific cluster of biological diversity that may contribute to the overall regional bacterial community functionality and furthermore increase the level of resilience to environmental change of the entire system [30].

Along the transect, the soil fraction communities were significantly different (PERMANOVA, df = 2.55; *F* = 2.70; *P* = 0.03). In particular the rhizosphere community, that resides in the first millimeter of soil adhering to the root, appeared completely different from the root surrounding soil (PERMANOVA, *t* = 2.04; *P* = 0.017, p-pht) and bulk soil (PERMANOVA, *t* = 2.05; *P* = 0.019, p-pht), suggesting the influence of palm root exudates in shaping the bacterial community. Generally, the rhizosphere is the transition zone between the root surface and soil where the released exudates and the rhizodeposition favour microbial proliferation, inducing changes in the structure and in the chemical-physical properties of the soil [31]. Indeed, the analysis of bacterial abundance in the rhizosphere showed a numbers of 16S rRNA copies ranging from 5.88 ± 0.78 to 6.63 ± 0.15 (Figure 1(d)). Despite similar values observed in the rhizosphere community, a statistical difference among the stations was identified (PERMANOVA, df = 6.20; *F* = 2.93; *P* = 0.041), mainly influenced by environmental factor directly linked to location, such as altitude and temperature maximum (DistLM, *P* = 0.03).

Despite the rhizosphere effect observed along the transect, in each oasis considered separately from the others, rhizobacterial community appeared directly connected to that present in the root surrounding soil and the bulk soil, since no statistically significant differences in the bacterial diversity were observed among the different soil fractions within each station (R, S e B: PERMANOVA, df = 12.55; *F* = 1.62; *P* = 0.057). The rhizosphere effect is particularly noticeable in nutrient-poor soils and under severe abiotic stresses, as previously observed for herbaceous and arboreal plants grown in arid lands [7, 8, 32, 33]. In the oasis model the selection mediated by “oasis ecosystem” appeared stronger than the one exerted by the plant root system (Supplementary Table 2). Naturally, most of the desert microbial communities seem to be structured solely by abiotic processes [34, 35]. However, desert farming may strongly affect the sand/soil microbial diversity reshaping the structure of the resident microbial communities [8, 9, 36, 37]. During long-term desert farming land management, such as that occurring in the studied oases, the structure of rhizosphere bacterial community is strongly influenced by the plant and the desert farming practices that determine drastic shifts in the composition of the original desert soil communities [7, 8].

Dist LM multivariate analysis was performed in order to correlate the differences in the structure of bacterial communities in the different oases with environmental parameters. The selection of soil microorganisms by the rhizosphere is a complex process controlled by several factors, often not easily correlated to the environmental settings [38]. Nevertheless, Dist LM analysis showed that geoclimatic parameters contributed to drive the assemblage of the bacterial communities. In particular, marginal test showed latitude, longitude, altitude, minimum temperature, and minimum rainfall as significant variables singularly involved in the selection of bacterial assemblages (Table 1(a)). Sequential test confirmed latitude, altitude, and temperature as variables involved in the bacterial community shaping (Table 1(b)). We can assume that a concurrence of environmental factors, including a hot and dry climate, may influence the differences among the bacterial communities of the soil fractions (R, S, and B) associated with the root system of date palm cultivated in the oases in the north and south macroregions examined (Figure 1(c)).

### 3.2. Cultivable Bacterial Communities Associated with Date Palm Soil Fractions

The isolation of native bacterial species associated with date palm root soil was performed using nonspecific media, in order to select a wide range of genera of possible plant growth promoters [39–41].

A total of 440 isolates were retrieved from the seven analyzed stations. To manage such a large set of isolates, total DNA was extracted from each isolate and 16S–23S rRNA gene internal transcribed spacers (ITS) were amplified. ITS characterization represents a useful molecular tool for the discrimination of bacterial isolates up to the subspecies level [42–45]. Within the whole bacterial collection, ITS-PCR fingerprinting revealed 30 distinct haplotypes (H1-H30). Haplotypes H4 and H20 were the most frequent and were represented by 46 and 26 isolates, respectively. Representative isolates (from one to four strains for each haplotype, summing up a total of 98 isolates) were subjected to species identification using partial 16S rRNA gene sequencing (Supplementary Figure 1).

**Figure 2:**
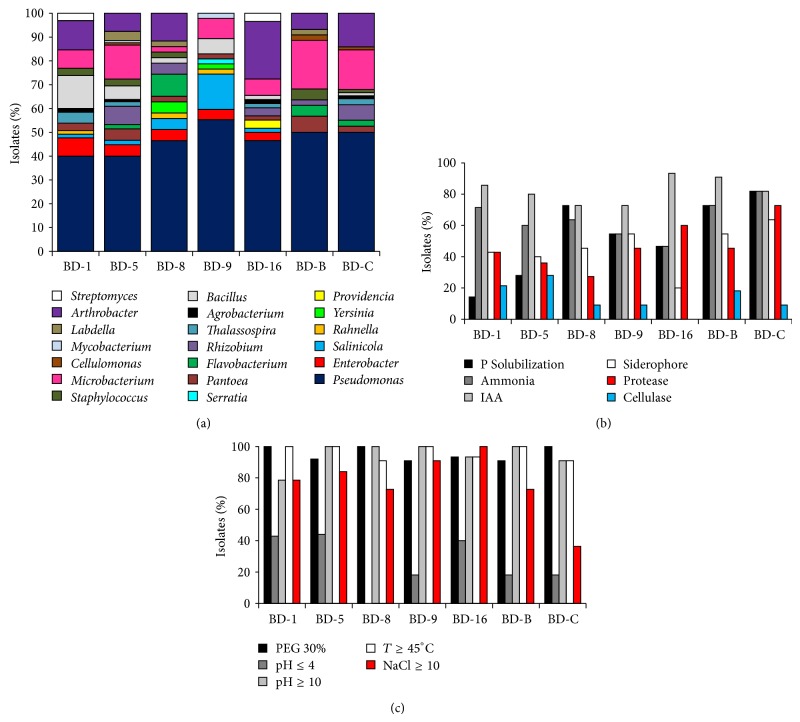
Diversity and functionality of cultivable bacteria islated from date palm rhizosphere. (a) Phylogenetic identification at the genus level of culturable bacteria associated with date palm rhizosphere. (b) Percentage of date palm rhizosphere-associated bacteria showing PGP activity. (c) Percentage of isolates displaying the assayed abiotic stress tolerance in the bacterial collection of strains associated with date palm cultivated in the seven oases analysed.

A wide diversity was detected into date palm rhizosphere bacterial community along the studied aridity transect in Tunisia. Significant differences were observed in the structure of the bacterial communities in the rhizosphere of the analyzed oases, in particular for the differential distribution pattern of the major bacterial genera (Figure 2(a)). According to the cluster analysis at the genus level performed on the entire strain collection, the composition of the cultivable rhizobacterial communities associated with date palm in the seven oases shared about the 65% similarity.

The phylogenetic identification of cultivable bacteria highlighted a predominance of gram-negative bacteria (66%), belonging to the* Gammaproteobacteria* (57%),* Alphaproteobacteria* (7%), and* Betaproteobacteria* (1%) subclasses. The remaining isolates were affiliated to the* Firmicutes* (7%),* Actinobacteria* (26%), and* Bacteroidetes* (2%) classes. Members of these taxa are frequently associated with different plant species and types, confirming that soil is the main reservoir of plant-associated bacteria [46]. The strains were allocated into 20 different genera of variable occurrence (Figure 2(a)), showing a high genetic diversity in the date palm rhizosphere presumably influenced by the combined effects of root exudates and agricultural management practices, particularly important under the arid pedoclimatic conditions [47]. The rhizobacterial communities were dominated by* Pseudomonas*, as previously described in herbaceous plants, arboreal and plant adapted to arid climates [11, 48–50].

Together with* Pseudomonas*,* Pantoea* and* Microbacterium* genera were observed in all stations followed by* Bacillus* and* Arthrobacter*, which were reported in six out of seven stations. As well,* Enterobacter*,* Salinicola*,* Rhizobium*, and* Staphylococcus* were recorded among 5 stations, suggesting the adaptation of these genera to the oasis environment. Except* Labedella*, the genera found in association with date palm rhizosphere have been previously recognized as being capable of colonizing plant root systems in arid environment [11, 38, 48, 50–52].

### 3.3. Characterization of Rhizobacteria PGP Potential

The plant microbiome is a key determinant of plant health and productivity. Plant-associated microbes can help plants stimulate growth, promote biotic and abiotic stress resistance and influence crop yield and quality by nutrient mobilization and transport [6]. While the possibility to contribute to control biotic stresses by plant-associated microorganisms is well characterized, less is known for abiotic stress. However, several promising examples of stress protecting bacteria are already reported in the literature [7, 38, 53]. Recent works demonstrated that drought-exposed plants cultivated under desert farming are colonized by bacterial communities with high PGP potential [7, 8]. Such a PGP potential can promote increased tolerance to water shortage, mediated by the induction of a larger root system (up to 40%) that enhances water uptake [7]. To assess if the oasis date palm PGP potential was conserved in the rhizosphere soil, 98 isolates were evaluated for a series of PGP traits. The majority (85%) of isolates showed multiple PGP activities, which may promote plant growth directly, indirectly, or synergistically. Only 15% of the rhizobacteria showed one or no activity, while no strains displayed all the screened PGP activities. The most common PGP trait was auxin production (83%), followed by ammonia synthesis (63%) and biofertilization activities such as solubilization of phosphates (48%) and siderophore production (44%). In our rhizobacterial collection, the IAA production was equally distributed among the seven oases selected along the aridity transect (Figure 2(b)), similarly to previous observations in other arboreal plants cultivated along a latitude transect [11], confirming that IAA synthesis is a widespread PGP trait. The IAA production ranged from 2.5 to 85 *μ*g/mL with 49% of the strains producing an amount ranging from 10 to 20 *μ*g/mL and the 38% showing higher levels of IAA (more than 20 *μ*g/mL). As already described in the literature,* Pseudomonas*,* Bacillus*,* Pantoea*,* Staphylococcus*, and* Microbacterium* were the most abundant taxa implicated in IAA production [48, 54, 55]. The high frequency of IAA producing strains suggests a role of PGP bacteria in contributing to regulate the root surface extension and consequently the potential of water and nutrient uptake [56].

Phosphorus, together with iron and nitrogen, is a key nutrient for plant, particularly in oasis soil where the availability of nutrient sources of animal origin is scarce [57]. The ability of rhizobacteria to solubilize phosphate (48%) through the production of organic acids or phytases can be very important in arid ecosystems [58, 59]. Strains of* Pantoea*,* Enterobacter*,* Pseudomonas*,* Streptomyces*, and* Rhizobium* genera were the most efficient solubilizers, as previously showed in other arid contexts such as Tunisian grapevine [48] and different crops in Bolivia [60]. Several siderophore-producing bacteria were observed in the rhizosphere (44%) probably because this PGP trait confers competitive colonization ability in iron-limiting soils. Iron is made available for the plant host and consequently exerts a biocontrol role reducing iron-dependent spore germination of fungi [61]. The siderophore-producing bacteria belonged mainly to the* Pseudomonas* genus (67%), followed by* Bacillus* (7%) and* Pantoea* (7%). Predominance of siderophore release by* Pseudomonas* bacteria was already reported in the rhizosphere of other plants [62, 63]. In addition to siderophore production, cell wall degrading enzymes implicated in fungal inhibition and the organic matter turnover [51] were investigated. The 49% and 15% of the examined isolates were able to produce proteases and cellulases, respectively, with the most active strains belonging to* Serratia marcescens* and* Sinorhizobium meliloti*, respectively [64, 65]. Ammonia production can indirectly affect plant growth through nitrogen supply [66]. This trait was represented in 64% of the isolates, confirming its spread in the palm-bacteria association.

Further analyses were performed to evaluate the adaptability of isolates to abiotic stresses (Figure 2(c)). Drought stress resistance was presented by 95% of the strains that could grow in presence of increasing concentrations of PEG. Most of the strains (98%) were able to grow at 45°C, while only 39% at 50°C. The capacity to tolerate high temperature drastically decreased (5%) at 55°C and only* Bacillus* and* Pseudomonas* strains showed this ability [67, 68]. Moderate halotolerance was presented by 75% of the isolates, while 50% tolerated up to 15% NaCl, 20% actively grew in presence of 20% NaCl, and only the 6% were extremely halotolerant (25% NaCl), indicating salinity as a major selective factor for the bacterial microbiomes in the Tunisian date palm oases. The formulation of halotolerant PGPR could be an interesting alternative for agriculture productivity in the oasis [69]. The tested rhizobacteria could grow in a wide pH range. Within the bacterial collection 96% and the 75% of the strains were facultative alkalophiles able to grow in basic media (up to pH = 12), while 34% of them could grow in acidic media (pH = 4) and only 6% was facultative acidophiles growing down to pH = 3.

## 4. Conclusion

Date palm represents the key plant species in desert oases being essential in determining the oasis microclimate that can allow agriculture. Palm exerts both physical and functional services involved in the creation of ideal condition for desert farming. Palm root system and rhizosphere soil showed a complex diversity that enclosed a reservoir of PGP bacteria involved in the regulation of plant homeostasis. Future work is needed to perform experiment about the ability of selected bacterial isolates in promoting plant growth under greenhouse and field conditions. In this context, the selection of autochthonous bacteria, together with the desert farming practices, could have promising perspectives for sustainable agriculture in oasis ecosystem.

## Supplementary Material

Supplementary material contains additional information about the geo-climatic data and biodiversity of bacterial community in the different studied oases.

## Figures and Tables

**Table 1a:** (a) Marginal test

Variable	SS (trace)	*F*	*P*
Lat. N	**1855.5**	**6.4211**	**0.0028**
Long. E	**1444.3**	**4.8698**	**0.0097**
Alt (m)	**980.15**	**3.2116**	**0.0376**
*T*_min⁡_ (°C)	**1170.8**	**3.8811**	**0.0193**
*T*_max⁡_ (°C)	363.14	1.147	0.3034
Rainfall min (mm)	**1187.3**	**3.9401**	**0.0198**
Rainfall max (mm)	588.06	1.8821	0.136

**Table 1b:** (b) Sequential test

Variable	AIC	SS	*F*	*P*	Cumul.	Res. df
(+) Lat. N	**319.28**	**1855.5**	**6.4211**	**0.0022**	**0.10627**	**54**
(+) Long. E	320.07	331.89	1.1517	0.2998	0.12528	53
(+) Alt (m)	**317.33**	**1240.6**	**4.5974**	**0.0122**	**0.19633**	**52**
(+) *T*_min⁡_ (°C)	316.7	644.65	2.4558	0.084	0.23325	51
(+) *T*_max⁡_ (°C)	**309.31**	**2066**	**9.1244**	**0.0006**	**0.35158**	**50**
(+) Rainfall min (mm)	**303.19**	**1528.3**	**7.6468**	**0.001**	**0.43911**	**49**
(+) Rainfall max (mm)	303.19	< 0.01	0	1	0.43911	49
