# Supplementary material for: The Date Palm Tree Rhizosphere Is a Niche for Plant Growth Promoting Bacteria in the Oasis Ecosystem
Source: Biomed Res Int. 2015 Mar 19;2015:153851. doi: 10.1155/2015/153851 (PMC4383278; doi:10.1155/2015/153851)
Supplement: Supplementary file 1 — Supplementary material contains additional information about the geo-climatic data and biodiversity of bacterial community in the different studied oases. [file 153851.f1.doc]

**SUPPLEMENTARY MATERIALS**

**Supplementary Figure 1. Phylogenetic affiliation of date palm rhizobacteria.** Neighbour-joining phylogenetic tree based on 16S rRNA gene sequences of rhizospheric bacteria and their closest phylogenetic neighbors. Bootstrap values are indicated at nodes. Scale bar represents observed number of changes per nucleotide position.

**
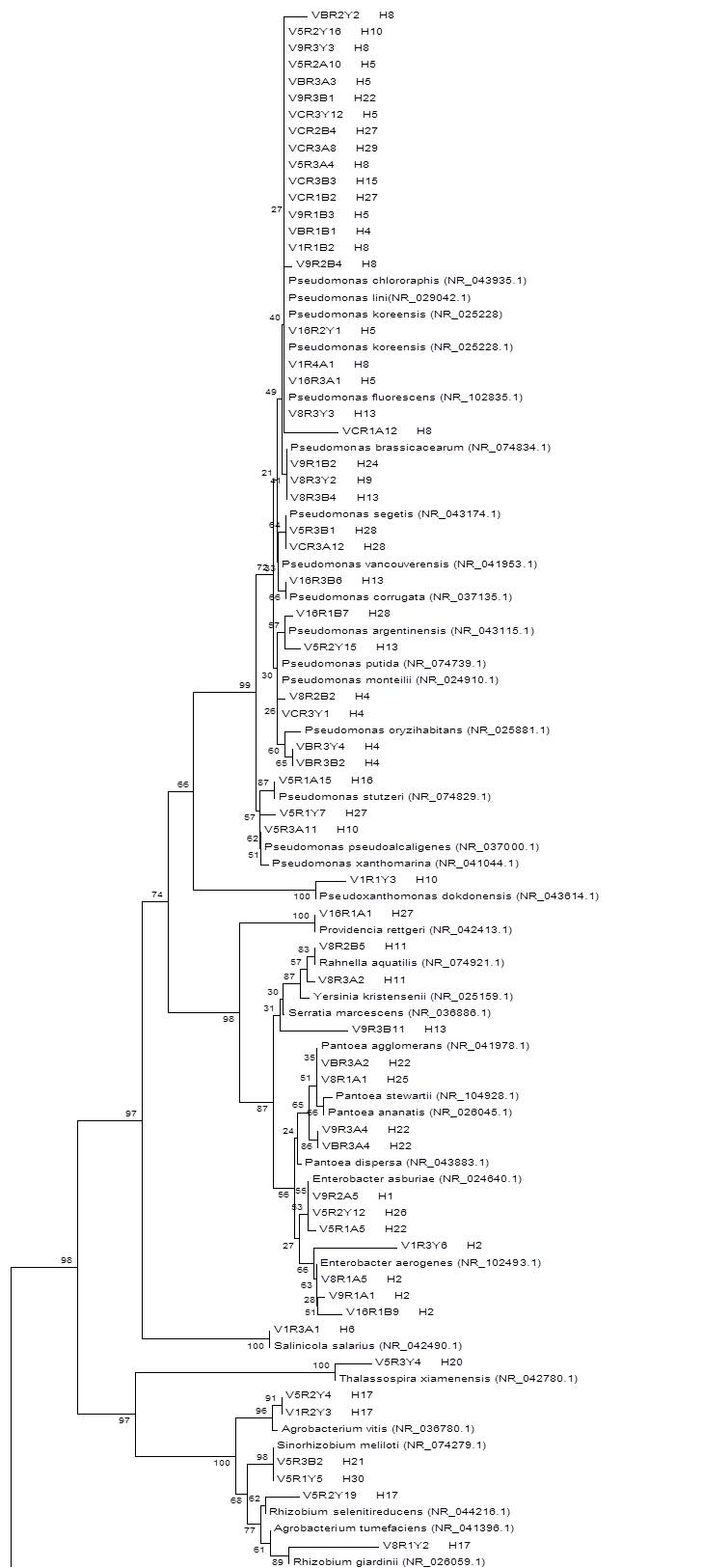
**

**Supplementary table 1.** Geo-climatic factors associated with the seven oases sampled in the south of Tunisia.

| **Station** | **Fraction** | **Oasis of origin** | **Latitude** | **Longitude** | **Altitude (m)** | **T min (°C)** | **T max (°C)** | **Rainfall min (mm)** | **Rainfall max (mm)** | **Aquifer** |
| --- | --- | --- | --- | --- | --- | --- | --- | --- | --- | --- |
| BD-1 | R, S, B | Ksar Ghilan | N32°59'012" | E09°38'072" | 210 | 5.4 | 39.4 | 0 | 13 | CI |
| BD-5 | R, S, B | Douz | N33°26'753" | E09°00'814" | 53 | 4.8 | 40.5 | 0 | 11 | CT |
| BD-8 | R, S, B | El Faouar | N33°21'613" | E08°41'780" | 35 | 4.3 | 39.3 | 0 | 16 | CT |
| BV-9 | R, S, B | Rejim Maatoug | N33°19'345" | E08°01'647" | 43 | 4.7 | 38.1 | 0 | 19 | CT |
| BD-16 | R, S, B | Tozeur | N34°02'585" | E08°12'777" | 36 | 4.5 | 40 | 0 | 14 | CT |
| BD-B | R, S, B | Tamerza | N34°23'00" | E07°56'08" | 285 | 4 | 38.5 | 2 | 18 | CT |
| BD-C | R, S, B | Ain el Karma | N34°24'15" | E07°59'00" | 386 | 4 | 38.5 | 2 | 18 | CT |

T= temperature; CI= continental intercalaire aquifer; CT= complex terminal aquifer Miocene and Plio-quaternaire.

**Supplementary table 2.** Statistical analysis of bacterial assemblage in function of environment, station and soil fraction. df= degrees of freedom; F= statistic F; p= probability (in bold the variables statistically significant; p<0.05).

| Factors | df | MS | F | p |
| --- | --- | --- | --- | --- |
| **Environment** | **1** | **1681** | **8.0657** | **0.0017** |
| **Station (Environment)** | **5** | **800.94** | **3.8430** | **0.0001** |
| Soil fraction (Station (Environment)) | 12 | 338.58 | 1.6245 | 0.0573 |
| Res | 37 | 208.42 |  |  |
| Total | 55 |  |  |  |

**Supplementary Table 3.** Pairwise statistical analysis of bacterial assemblages across locations**.** t= t-student; p= probability (in bold the variables statistically significant; p<0.05).

| **Location pairs** | **t** | ***p*** |
| --- | --- | --- |
| Ksar Ghilan vs Douz | 1.6151 | 0.1833 |
| Ksar Ghilan vs El Faouar | 1.5229 | 0.1992 |
| Ksar Ghilan vs Rejim Maatoug | 1.4970 | 0.2103 |
| Ksar Ghilan vs Tozeur | 1.4977 | 0.2023 |
| Ksar Ghilan vs Tamerza | 0.2070 | 0.8604 |
| Ksar Ghilan vs Ain el karma | 0.8580 | 0.4363 |
| Douz vs El Faouar | 0.6298 | 0.5555 |
| Douz vs Rejim Maatoug | 0.5765 | 0.5950 |
| Douz vs Tozeur | 0.6633 | 0.5444 |
| **Douz vs Tamerza** | **3.2046** | **0.0335** |
| Douz vs Ain el karma | 1.3743 | 0.2395 |
| El Faouar vs Rejim Maatoug | 0.0600 | 0.9576 |
| El Faouar vs Tozeur | 0.2408 | 0.8197 |
| **El Faouar vs Tamerza** | **12.913** | **0.0001** |
| El Faouar vs Ain el karma | 1.6755 | 0.1702 |
| Rejim Maatoug vs Tozeur | 0.1472 | 0.8943 |
| **Rejim Maatoug vs Tamerza** | **6.5911** | **0.0032** |
| Rejim Maatoug vs Ain el karma | 1.4559 | 0.2102 |
| **Tozeur vs Tamerza** | **11.866** | **0.0006** |
| Tozeur vs Ain el karma | 1.5960 | 0.1869 |
| **Tamerza vs Ain el karma** | **2.8786** | **0.0486** |
